# Infection prevention and control compliance in Tanzanian outpatient facilities: a cross-sectional study with implications for the control of COVID-19

**DOI:** 10.1016/S2214-109X(20)30222-9

**Published:** 2020-05-06

**Authors:** Timothy Powell-Jackson, Jessica J C King, Christina Makungu, Nicole Spieker, Susannah Woodd, Peter Risha, Catherine Goodman

**Affiliations:** a Department of Global Health and Development, London School of Hygiene & Tropical Medicine, London, UK; b Department of Infectious Disease Epidemiology, London School of Hygiene & Tropical Medicine, London, UK; c Ifakara Health Institute, Dar es Salaam, Tanzania; d PharmAccess, Amsterdam, Netherlands; e PharmAccess Tanzania, Dar es Salaam, Tanzania

## Abstract

**Background:**

As coronavirus disease 2019 (COVID-19) spreads, weak health systems must not become a vehicle for transmission through poor infection prevention and control practices. We assessed the compliance of health workers with infection prevention and control practices relevant to COVID-19 in outpatient settings in Tanzania, before the pandemic.

**Methods:**

This study was based on a secondary analysis of cross-sectional data collected as part of a randomised controlled trial in private for-profit dispensaries and health centres and in faith-based dispensaries, health centres, and hospitals, in 18 regions. We observed provider–patient interactions in outpatient consultation rooms, laboratories, and dressing rooms, and categorised infection prevention and control practices into four domains: hand hygiene, glove use, disinfection of reusable equipment, and waste management. We calculated compliance as the proportion of indications (infection risks) in which a health worker performed a correct action, and examined associations between compliance and health worker and facility characteristics using multilevel mixed-effects logistic regression models.

**Findings:**

Between Feb 7 and April 5, 2018, we visited 228 health facilities, and observed at least one infection prevention and control indication in 220 facilities (118 [54%] dispensaries, 66 [30%] health centres, and 36 [16%] hospitals). 18 710 indications were observed across 734 health workers (49 [7%] medical doctors, 214 [29%] assistant medical officers or clinical officers, 106 [14%] nurses or midwives, 126 [17%] clinical assistants, and 238 [32%] laboratory technicians or assistants). Compliance was 6·9% for hand hygiene (n=8655 indications), 74·8% for glove use (n=4915), 4·8% for disinfection of reusable equipment (n=841), and 43·3% for waste management (n=4299). Facility location was not associated with compliance in any of the infection prevention and control domains. Facility level and ownership were also not significantly associated with compliance, except for waste management. For hand hygiene, nurses and midwives (odds ratio 5·80 [95% CI 3·91–8·61]) and nursing and medical assistants (2·65 [1·67–4·20]) significantly outperformed the reference category of assistant medical officers or clinical officers. For glove use, nurses and midwives (10·06 [6·68–15·13]) and nursing and medical assistants (5·93 [4·05–8·71]) also significantly outperformed the reference category. Laboratory technicians performed significantly better in glove use (11·95 [8·98–15·89]), but significantly worse in hand hygiene (0·27 [0·17–0·43]) and waste management (0·25 [0·14–0·44] than the reference category. Health worker age was negatively associated with correct glove use and female health workers were more likely to comply with hand hygiene.

**Interpretation:**

Health worker infection prevention and control compliance, particularly for hand hygiene and disinfection, was inadequate in these outpatient settings. Improvements in provision of supplies and health worker behaviours are urgently needed in the face of the current pandemic.

**Funding:**

UK Medical Research Council, Economic and Social Research Council, Department for International Development, Global Challenges Research Fund, Wellcome Trust.

## Introduction

The coronavirus disease 2019 (COVID-19) pandemic is likely to spread to most, if not all, countries globally.^1^ As of April, 2020, the exponential growth phase has been concentrated in high-income or upper-middle-income countries, but the virus could represent a huge threat to lower-income countries in the months to come. The high risks in these countries reflect a combination of country characteristics: densely populated urban areas, high rates of self-employment with no sick pay, poor community hygiene and sanitation, and weak health systems.^2^ These health systems lack adequate surveillance and laboratory capacity^3^ and a sufficient supply of appropriately trained health workers, and have utterly insufficient critical care capacity to address the upsurge in severe COVID-19 cases^4^—challenges that also severely hampered the response to Ebola outbreaks.^5^, ^6^ While dealing with this surge in cases, it is essential that the health systems themselves do not become a vehicle for transmission to patients or front-line health workers.

**Research in context**

**Evidence before this study**

We searched for studies in English that had investigated health worker compliance with infection prevention and control practices in a low-income country using structured observations, from Jan 1, 2010, to April 14, 2020, using PubMed. The search strategy included terms related to infection prevention and control (“infection prevention”, “infection control”, “IPC”, or “hand hygiene”), the clinical setting (“outpatient”, “primary care”), and the country setting (full list of low-income and middle-income country [LMIC] names, full list of standard terms for LMIC setting). Three studies met the inclusion criteria. A study of 945 health facilities in Kenya measured health worker compliance across five domains of infection prevention and control in 2015. It found that compliance was 2·3% for hand hygiene, 41·0% for glove practices, 87·1% for injections and blood samples, 14·7% for reusable equipment, and 5·4% for non-sharp waste segregation. A second study showed hand hygiene compliance of 38·9% in one teaching hospital in Jamaica. Another study examining injection practices in primary care in Bangladesh showed hand hygiene compliance to be 5·6%, with new syringes and needles used 84·5% of the time and correctly disposed of 18·5% of the time.

**Added value of this study**

To our knowledge, this is one of very few large-scale studies to examine health worker compliance with infection prevention and control practices during outpatient care in a low-income country. In this study of 220 private for-profit and faith-based health facilities, conducted in 2018, we observed a total of 18 710 infection prevention and control indications relevant to coronavirus disease 2019 (COVID-19) across 3688 provider–patient interactions involving 734 different health workers. Infection prevention and control compliance was poor overall, regardless of facility level, ownership, or location. Compliance was 6·9% for hand hygiene, 74·8% for glove use, 4·8% for disinfection of reusable equipment, and 43·3% for waste management. Nurses and midwives performed better than more highly qualified health workers for hand hygiene and glove use.

**Implications of all the available evidence**

The available evidence shows that compliance with infection prevention and control practices will need to improve dramatically if the health systems in east Africa are going to contain rather than fuel the transmission of COVID-19. The findings highlight the urgent need for policy makers to address very low baseline infection prevention and control compliance in health facilities. These findings should inform strategies designed to increase supplies needed for infection prevention and control and to influence the behavioural determinants of compliance with the relevant practices.

It is thought that the two main routes of transmission for severe acute respiratory syndrome coronavirus 2 (SARS-CoV-2), the virus responsible for COVID-19, are through respiratory droplets and through contact. When someone infected with SARS-CoV-2 coughs or exhales, they produce infective respiratory droplets that can be inhaled by anyone close to them. In addition, droplets landing on nearby surfaces can be a source of contact transmission via the hands to the nose, mouth, and eyes.^7^ SARS-CoV-2 remains viable on surfaces for 4–72 h, depending on the surface, similar to other human coronaviruses.^8^, ^9^ Therefore, appropriate infection prevention and control in health facilities is crucial, particularly given asymptomatic transmission^10^, ^11^ and the possible increase in treatment seeking as morbidity increases. Whereas in many high-income countries the advice has been not to visit a health facility if you have fever or cough, in low-income and middle-income countries (LMICs), this advice might be much less appropriate given the high prevalence of other potentially serious diseases with similar symptoms, such as malaria, pneumonia, HIV, and tuberculosis. Moreover, it is uncertain how well such advice would be heeded or enforced. In the context of poor infection prevention and control practices, the use of outpatient services by people with COVID-19 could lead to high rates of infection in health workers, and could fuel health-care-associated infections, which are already prevalent in LMIC settings.^12^

WHO recently issued an urgent interim guidance document on water, sanitation, hygiene, and waste management for COVID-19, stressing the importance of these practices in health-care settings.^7^ The guidance encouraged frequent and proper hand hygiene as one of the most important measures to prevent infection with SARS-CoV-2, and emphasised regular cleaning and disinfection practices, and safe management of health-care waste and excreta. This guidance builds on and further underscores existing standard infection prevention and control guidelines for health facilities.^13^, ^14^

We assessed compliance with infection prevention and control practices in outpatient settings, drawing on a secondary analysis of data from 220 health facilities, originally collected in 2018 to evaluate the effectiveness of a quality-improvement intervention in 18 of the 22 regions of mainland Tanzania. Among all the infection prevention and control practices measured, we focused on those most relevant for COVID-19 transmission: hand hygiene, glove use, disinfection of reusable equipment, and waste management. These data show the situation before the pandemic, but have the potential to inform strategies and interventions needed to contain transmission. We further explored associations between compliance with infection prevention and control practices and the characteristics of health facilities and health workers.

## Methods

### Study design and setting

We used a cross-sectional study design to examine compliance with infection prevention and control practices in a large sample of health workers located in faith-based and private for-profit health facilities across mainland Tanzania. We drew on secondary data collected as part of a cluster-randomised controlled trial of a quality-improvement programme (ISRCTN93644888).

Tanzania is a low-income country that had a gross domestic product per capita of US$1051 and a population of 56 million in 2018.^15^ It has a mixed health system, with a growing private health-care sector. Faith-based facilities have long been important health-care providers, often closely integrated with the public health system, and typically having some government-salaried staff. Private for-profit facilities have steadily grown in number, especially in the past decade. Although the public sector remains the dominant health-care provider, the private share is sizeable: in 2019, nationally, 13% of providers were private for-profit and 12% were faith-based, while in urban areas the private shares were much higher (52% for-profit and 19% faith-based in the city of Dar es Salaam).^16^ Facilities are categorised by level: dispensaries (the lowest level, often staffed by a single clinical officer with 3 years of post-secondary education), health centres (larger facilities with more staff, which might admit some patients), and hospitals (which have inpatient wards and usually have a fully qualified doctor).

Study facilities were recruited from March 7 to Nov 30, 2016, by two partners: the Association of Private Health Facilities in Tanzania (APHFTA), which represents mainly for-profit facilities; and the Christian Social Services Commission (CSSC), which represents most mission facilities. Facilities were recruited from the Northern, Eastern, Central, Southern, and Southern Highlands zones. At the time of recruitment there were 462 APHFTA member facilities and 513 CSSC member facilities in the study zones. We selected a non-random list of 280 potentially eligible facilities for participation. APHFTA and CSSC approached these facilities to confirm eligibility and obtain written informed consent. Of the facilities approached, 43 were found to be ineligible or were unwilling to participate, giving a total of 237 facilities participating in the randomised controlled trial. For the current study, we used data from the endline sample. Using an endline sample from the quality-improvement programme evaluation raises the question of whether these facilities had higher infection prevention and control compliance than would normally be expected. However, compliance was very similar between intervention and control groups at endline, with no significant difference between the two groups (the results of the randomised controlled trial will be reported elsewhere).

The study protocol was approved by the ethics committees of the Ifakara Health Institute (approval number 04-2016) and the National Institute of Medical Research (IX/2415) in Tanzania, and the London School of Hygiene & Tropical Medicine (10493) in the UK. Reporting in this Article follows the STROBE guidelines for observational studies.^17^

### Participants

Eligible facilities were dispensaries and health centres that were APHFTA members, and dispensaries, health centres, and hospitals that were CSSC members. Facilities were ineligible if they refused consent, provided specific services only (eg, mental health or maternity services), or were tertiary hospitals. We obtained written consent from each health worker observed. Patients were eligible for observation if they (or their adult caretaker if younger than 18 years of age) gave verbal informed consent.

### Data collection

Data were collected through a facility survey and through observations of infection prevention and control practices, both done during the same health facility visit.

We used clinical observations and a tool adapted from a study by Bedoya and colleagues,^18^ itself based on WHO guidelines,^13^, ^19^ to measure infection prevention and control compliance in health workers. The assessment was based on the concept of indications (ie, moments in a provider–patient interaction that present an infection risk to either patient, provider, or both). For example, if the provider takes the patient's temperature with a non-infrared thermometer, a patient is exposed to an infection risk. For every indication, there is a corresponding action. In the case of a thermometer, a corresponding action is disinfecting the thermometer between patients with rubbing alcohol or bleach.

Fieldworkers spent 6 h in each facility observing interactions in outpatient consultation rooms, laboratories, and injection or dressing rooms. A long-standing concern with clinical observations is the Hawthorne effect, in which study subjects' awareness of being observed causes them to alter their behaviour.^20^ To minimise such bias, fieldworkers were coached to observe discreetly from the corner of the room, limit interaction with either provider or patient, and not disclose that observations were focused on infection prevention and control.

We used a structured tool during interviews with the manager in charge to obtain information on health facility characteristics. We also took a roster of every health worker present in the outpatient department on the day of the visit, covering their characteristics. A facility assessment done by APHFTA and CSSC 2–4 months after our infection prevention and control observations collected information on the availability of some infrastructure and supplies.

### Variables

The tool assessing infection prevention and control compliance specified 20 indications and corresponding actions (appendix pp 4–5). In this analysis, we primarily focused on the 15 indications most relevant to COVID-19. These indications and the corresponding actions can be grouped into four domains: hand hygiene, glove use, disinfection of reusable equipment, and waste management (table 1). We defined compliance as a binary variable which took a value of one if the correct action was taken in response to an indication, and zero otherwise. We also assessed a further five indications on injection and blood draw safety, for which we report compliance in the appendix (p 6), since they are not directly related to COVID-19. Health facility characteristics included the following categories: facility level, facility ownership, and facility location. Characteristics of the health workers observed included age, gender, and cadre.

**Table 1:** Definitions of infection prevention and control indications and their corresponding actions

	**Action for compliance**
**Hand hygiene domain**	
Before touching a patient	Provider washed hands with soap or used alcohol hand rub and did not dry hands on reused towel or clothes
After touching a patient	As above
Before a clean or aseptic procedure	As above
After exposure to body fluids	As above
Before injection or blood draw	As above
After injection or blood draw	As above
**Glove use domain**	
When carrying out intravenous injection, blood draw, wound cleaning, or dressing	Gloves used
For any other contact with body fluid, mucous membranes, or non-intact skin	Gloves used
When using gloves	New gloves were used for each patient
**Disinfection of reusable equipment domain**	
Before or after use of non-infrared thermometer	Disinfected using rubbing alcohol or bleach
Before or after use of otoscope	As above
Before or after use of stethoscope	As above
**Waste management domain**	
After using gloves	Gloves discarded into waste bin
After injection or blood draw that produced non-sharp infectious waste	Swabs, cotton wool, test strips, and capillary tubes segregated into red or yellow waste bin with matching bag, or safety or improvised sharps container
After a medical examination or procedure that produced infectious waste	Swabs, gauze, cotton wool, and disposal tongue depressors segregated into red or yellow waste bin with matching bag

### Statistical analysis

Analyses were done at the level of indication—ie, each observation in the dataset was an indication. We first analysed infection prevention and control practices descriptively, reporting compliance (as a percentage) by indication and domain. To examine variation in infection prevention and control compliance according to the characteristics of health facilities and health workers, we did bivariate and adjusted analyses. We did this separately for each of the four infection prevention and control domains by pooling the data on indications within a domain. In the adjusted analyses, we used models that accounted for the hierarchical nature of the data. Specifically, we ran multilevel mixed-effects logistic regressions that included facility random effects. These models also included, as fixed effects, the full set of facility and health worker characteristics, as well as indicators for each indication within the infection prevention and control domain.

We did several sensitivity analyses and robustness checks (appendix pp 2–3). First, we additionally included in the regression characteristics of the patient (age categories, gender) to adjust for patient mix. Second, we provided evidence on any Hawthorne effect by examining whether compliance with infection prevention and control practices was associated with order number of patients observed. Third, using data on the universe of health facilities in the country,^16^ we reweighted the data to account for the fact that we oversampled certain types of facility (ownership and level). All data were analysed using Stata/SE version 16.1.

### Role of the funding source

The funders of the study had no role in the study design, data collection, data analysis, data interpretation, or writing of the report. TP-J, JJCK, CM, and CG had full access to all the data in the study and all authors had final responsibility for the decision to submit for publication.

## Results

Of the 237 facilities participating in the randomised controlled trial, eight had permanently closed down and one was closed for renovations at endline. We visited the remaining 228 health facilities between Feb 7 and April 5, 2018, to observe infection prevention and control practices. We observed at least one provider–patient interaction in 223 facilities, and at least one infection prevention and control indication in 220 facilities. We observed a total of 18 710 indications relevant to COVID-19, in 5425 provider–patient interactions (of which 3688 had at least one indication), with 2840 unique patients and 734 unique health providers.

The facility sample included 118 (54%) dispensaries, 66 (30%) health centres, and 36 (16%) hospitals (table 2). Among the 733 observed health workers with available data, 338 (46%) were younger than 30 years, 403 (55%) were male, and 330 (45%) were female. 49 (7%) were fully qualified medical doctors (often referred to as medical officers), and 214 (29%) were assistant medical officers or clinical officers, who also work as clinicians but have lower qualifications. 106 (14%) were qualified nurses or midwives, and 126 (17%) were in a clinical assistant role (typically not requiring post-secondary education). 238 (32%) worked in the laboratory, as technicians or assistants. Table 2 also shows availability of key infection prevention and control materials.

**Table 2:** Facility and health worker characteristics

		**Participants**
Health worker characteristics (n=734)*		
Age, years		
	<30	338 (46%)
	30–49	242 (33%)
	≥50	153 (21%)
Sex		
	Male	403 (55%)
	Female	330 (45%)
Cadre		
	Medical doctor	49 (7%)
	Assistant medical officer or clinical officer	214 (29%)
	Nurse or midwife	106 (14%)
	Nursing or medical assistant	126 (17%)
	Laboratory technician or assistant	238 (32%)
**Facility characteristics (n=220)**†		
Facility level and ownership		
	Private for-profit dispensaries	79 (36%)
	Private for-profit health centres	19 (9%)
	Faith-based organisation dispensaries	39 (18%)
	Faith-based organisation health centres	47 (21%)
	Faith-based organisation hospitals	36 (16%)
Facility location		
	Dar es Salaam	42 (19%)
	Other urban or peri-urban location	89 (40%)
	Rural location	89 (40%)
**Facility availability of infrastructure and supplies (n=221)**‡		
Adequate hand hygiene facilities in outpatient consultation rooms§		
	Yes	27 (12%)
	Partially	149 (67%)
	No	45 (20%)
Health-care waste collection assets with colour-coded segregation¶		
	Yes	57 (26%)
	Partially	106 (48%)
	No	54 (24%)
Clean water supply in all essential areas		
	Yes	192 (87%)
	Partially	17 (8%)
	No	12 (5%)

Data are n (%).

* Age, sex, and cadre were missing for one observed health worker.

† Data are from our facility survey; eligible interactions were observed in 220 of the 228 facilities visited.

‡ Data are from a facility assessment done by the Association of Private Health Facilities in Tanzania and the Christian Social Services Commission 2–4 months after the infection prevention and control observations.

§ Water, soap, and single-use towels, or gel sanitisers available.

¶ Waste collection materials and units are available in all critical departments of the health-care facility and comply with the colour coding chart (ie, coloured bags or containers: black for non-infectious, yellow for infectious, and red for highly infectious sharps container).

**Figure 1:**
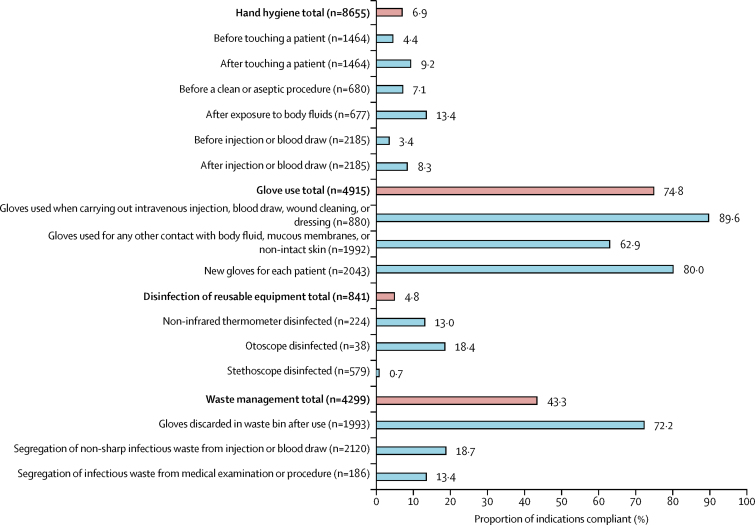
Compliance with infection prevention and control actions by indication and domain

Infection prevention and control compliance varied substantially by domain (figure 1). Health workers rarely adhered to hand hygiene practices (593 [6·9%] of 8655 indications) or disinfection of reusable equipment (40 [4·8%] of 841). By contrast, compliance was much higher for waste management (1860 [43·3%] of 4299) and glove use (3676 [74·8%] of 4915). In general, compliance was similar across indications within the same domain (eg, compliance was <14% for all hand hygiene indications, and >62% for all glove use indications). The indications with the lowest compliance were disinfection of stethoscopes (4 [0·7%] of 579), and hand hygiene before injection or blood draw (74 [3·4%] of 2185) and before touching a patient (65 [4·4%] of 1464). Hand hygiene was especially low for the subset of indications in this domain when health workers used gloves: 32 (1·6%) of 2064 indications before and 66 (3·6%) of 1854 after glove use (data not shown). Compliance with injection and blood draw safety was 10 378 (95·2%) of 10 897, and was above 90% in each of the five indications in this domain (appendix p 6).

**Figure 2:**
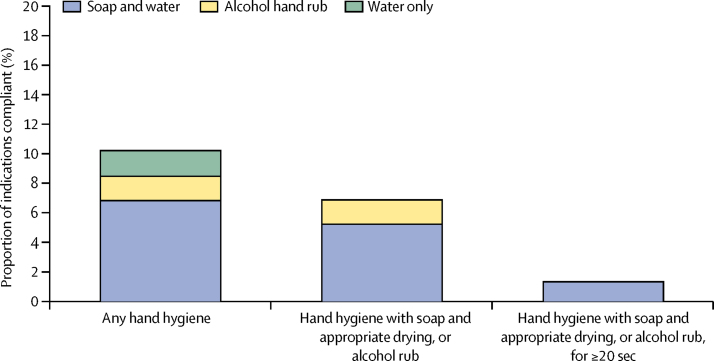
Compliance for hand hygiene indications (n=8655)

Figure 2 provides a more detailed breakdown of hand hygiene practices. Health workers attempted some hand hygiene in 883 (10·2%) of 8655 instances, but this was non-compliant in 151 (1·7%) instances because it involved only water (no soap or alcohol hand rub). There was non-compliance in an additional 139 (1·6%) instances because hands were dried on clothes or a reused towel after otherwise correct hand hygiene. This left 593 (6·9%) instances in which health workers complied with hand hygiene practices (450 [5·2%] soap, water, and appropriate hand drying; 143 [1·7%] alcohol rub). We also trained fieldworkers to estimate the duration of hand hygiene with soap and water or hand rub. As it was not possible for them to use a timing device unobtrusively, these estimates are approximate and were not included in the compliance definition in figure 1. If the compliance definition did require washing or hand rub application for 20 sec or more, hand hygiene compliance fell to 115 (1·3%) instances (figure 2).

Table 3 reports odds ratios (ORs) for the associations between compliance in each infection prevention and control domain and the characteristics of facilities and health workers. Facility level and ownership were not significantly associated with compliance in the domains of hand hygiene, glove use, and disinfection of reusable equipment. Faith-based health centres (OR 0·36 [95% CI 0·18–0·72], p=0·0037) and hospitals (0·46 [0·22–0·95], p=0·037) were less likely to comply with waste management practices than for-profit dispensaries after adjusting for other facility and health worker characteristics and indications. Further analysis suggested that the lack of matching coloured bags for waste bins drove this particular association (appendix p 13). Facility location was not associated with compliance in any of the infection prevention and control domains.

**Table 3:** Associations between compliance in each domain and facility and health worker characteristics

		**Hand hygiene (n=8651)**			**Glove use (n=4912)**			**Disinfection of reusable equipment (n=751)**			**Waste management (n=4296)**		
		Compliance, n/N (%)	OR (95% CI)	p value	Compliance, n/N (%)	OR (95% CI)	p value	Compliance, n/N (%)	OR (95% CI)	p value	Compliance, n/N (%)	OR (95% CI)	p value
**Facility characteristics**													
Facility level and ownership													
	Private for-profit dispensaries	169/2711 (6·2%)	1·00 (ref)	..	1129/1496 (75·5%)	1·00 (ref)	..	14/306 (4·6%)	1·00 (ref)	..	625/1272 (49·1%)	1·00 (ref)	..
	Private for-profit health centres	64/752 (8·5%)	1·68 (0·59–4·76)	0·326	275/385 (71·4%)	1·01 (0·47–2·17)	0·974	0/0	..	..	139/336 (41·4%)	0·74 (0·31–1·77)	0·498
	Faith-based organisation dispensaries	73/1166 (6·3%)	0·86 (0·35–2·17)	0·750	530/664 (79·8%)	1·64 (0·86–3·15)	0·133	4/124 (3·2%)	1·52 (0·06–36·0)	0·797	286/584 (49·0%)	0·74 (0·35–1·55)	0·420
	Faith-based organisation health centres	200/1958 (10·2%)	1·91 (0·83–4·39)	0·129	803/1131 (71·0%)	0·83 (0·46–1·52)	0·548	9/161 (5·6%)	1·76 (0·09–36·30)	0·713	363/993 (36·6%)	0·36 (0·18–0·72)	0·0037
	Faith-based organisation hospitals	86/2064 (4·2%)	0·87 (0·35–2·17)	0·768	937/1236 (75·8%)	1·32 (0·70–2·50)	0·384	13/160 (8·1%)	14·1 (0·48–378·96)	0·122	446/1111 (40·1%)	0·46 (0·22–0·95)	0·037
Facility location													
	Dar es Salaam	92/1413 (6·5%)	1·00 (ref)	..	629/792 (79·4%)	1·00 (ref)	..	7/140 (5·0%)	1·00 (ref)	..	338/687 (49·2%)	1·00 (ref)	..
	Other urban or peri-urban location	273/3952 (6·9%)	1·25 (0·55–2·80)	0·594	1632/2235 (73·0%)	0·73 (0·41–1·30)	0·279	19/318 (6·0%)	2·93 (0·16–52·42)	0·466	780/1940 (40·2%)	0·66 (0·34–1·26)	0·210
	Rural location	227/3286 (6·9%)	0·87 (0·35–2·21)	0·778	1413/1885 (75·0%)	0·62 (0·32–1·21)	0·162	14/293 (4·8%)	0·36 (0·01-12·3)	0·570	741/1669 (44·4%)	1·13 (0·54–2·38)	0·741
**Staff characteristics**													
Age, years													
	<30	239/3533 (6·8%)	1·00 (ref)	..	1893/2333 (81·1%)	1·00 (ref)	..	15/209 (7·2%)	1·00 (ref)	..	964/2125 (45·4%)	1·00 (ref)	..
	30–49	209/3031 (6·9%)	1·09 (0·81–1·46)	0·575	1379/1815 (76·0%)	0·64 (0·50–0·82)	0·0004	18/227 (7·9%)	2·44 (0·26–22·9)	0·436	715/1637 (43·7%)	0·83 (0·62–1·09)	0·182
	≥50	144/2087 (6·9%)	0·89 (0·63–1·26)	0·525	402/764 (52·6%)	0·33 (0·23–0·45)	<0·0001	7/315 (2·2%)	0·09 (0·01–1·51)	0·095	180/534 (33·7%)	0·74 (0·48–1·15)	0·185
Gender													
	Male	269/5474 (4·9%)	1·00 (ref)	..	1997/2780 (71·8%)	1·00 (ref)	..	30/627 (4·8%)	1·00 (ref)	..	985/2336 (42·2%)	1·00 (ref)	..
	Female	323/3177 (10·2%)	1·90 (1·45–2·50)	<0·0001	1677/2132 (78·7%)	0·89 (0·70–1·13)	0·342	10/124 (8·1%)	2·09 (0·19–22·5)	0·542	874/1960 (44·6%)	1·01 (0·77–1·33)	0·945
Cadre													
	Medical doctor	46/588 (7·8%)	1·19 (0·72–1·95)	0·500	34/117 (29·1%)	0·57 (0·32–1·02)	0·058	7/102 (6·9%)	1·11 (0·06–20·87)	0·945	18/40 (45·0%)	NS	NS
	Assistant medical officer or clinical officer	188/3054 (6·2%)	1·00 (ref)	..	182/558 (32·6%)	1·00 (ref)	..	31/635 (4·9%)	1·00 (ref)	..	102/163 (62·6%)	1·00 (ref)	..
	Nurse or midwife	167/751 (22·2%)	5·80 (3·91–8·61)	<0·0001	396/485 (81·6%)	10·06 (6·68–15·13)	<0·0001	0/0	..	..	244/450 (54·2%)	0·82 (0·44–1·52)	0·524
	Nursing or medical assistant	115/888 (13·0%)	2·65 (1·67–4·20)	<0·0001	468/616 (76·0%)	5·93 (4·05–8·71)	<0·0001	1/9 (11·1%)	NS	NS	266/590 (45·1%)	0·58 (0·32–1·06)	0·076
	Laboratory technician or assistant	76/3370 (2·3%)	0·27 (0·17–0·43)	<0·0001	2594/3136 (82·7%)	11·95 (8·98–15·89)	<0·0001	1/5 (20·0%)	NS	NS	1229/3053 (40·3%)	0·25 (0·14–0·44)	<0·0001

Results are from multilevel mixed-effects logistic regression models. Each of the four models adjusts for facility and patient characteristics (as reported), as well as an indicator for each indication within the infection prevention and control domain (not reported). OR=odds ratio. NS=not shown (fewer than 50 observations done).

Health worker age was negatively associated with correct glove use, with lower compliance among 30–49-year-olds (OR 0·64 [95% CI 0·50–0·82], p=0·0004) and those aged 50 years or older (0·33 [0·23–0·45], p<0·0001) compared with those younger than 30 years. Female health workers were more likely than male health workers to comply with hand hygiene (1·90 [1·45–2·50], p<0·0001). Age and gender were not associated with compliance in other domains. However, cadre was a strong predictor of compliance in three domains. Compliance with hand hygiene was significantly higher among nurses and midwives (5·80 [3·91–8·61], p<0·0001) and nursing and medical assistants (2·65 [1·67–4·20], p<0·0001) than among assistant medical and clinical officers (the reference category), while compliance among laboratory technicians and assistants was much lower (0·27 [0·17–0·43], p<0·0001). Compliance with glove use was significantly higher than the reference category for nurses and midwives (10·06 [6·68–15·13], p<0·0001), medical and nursing assistants (5·93 [4·05–8·71], p<0·0001), and laboratory staff (11·95 [8·98–15·89], p<0·0001), and was slightly but non-significantly lower among medical doctors (0·57 [0·32–1·02], p=0·058). Laboratory staff were significantly less likely to comply with waste management practices than were assistant medical and clinical officers (0·25 [0·14–0·44], p<0·0001).

In sensitivity analyses, the results did not change when we further adjusted for patient age and gender, and findings were qualitatively similar when we weighted the data to account for oversampling with respect to facility level and ownership (appendix pp 2–3). There was no strong evidence of a relationship between health worker compliance and the order number of the patients observed. In some specifications, there was a small negative association, but this disappeared when health worker fixed effects were included (appendix pp 2–3).

## Discussion

Compliance with infection prevention and control practices is essential for minimising transmission of infection and is particularly crucial during the COVID-19 pandemic. Using secondary data from a 2018 study, we found that, under typical circumstances in Tanzanian outpatient facilities, infection prevention and control compliance was inadequate. Of primary concern was inadequate hand hygiene, which WHO has cited as crucial for COVID-19 containment, and for which overall compliance was only 6·9%. Hand hygiene was particularly rarely practised when gloves were used, indicating that there was little understanding that contamination of hands could take place despite the use of gloves.^21^ Compliance was also extremely low for disinfection of reusable equipment—only 13% of thermometers and less than 1% of stethoscopes, items both likely to be very widely used for patients with COVID-19 symptoms, were disinfected between patients. Performance on management of (non-sharp) waste was also below 20%, which could mean that additional infection opportunities arise if hazardous waste materials are subsequently not disposed of appropriately. One might expect that infection prevention and control compliance will increase compared with these findings from 2018 as awareness of COVID-19 grows among health workers and communication campaigns are launched. However, it is also possible that compliance will worsen as facilities see far greater patient volumes, leading to greater pressure on health worker time and supplies needed for the prevention and control of infection.

The data were collected from 220 faith-based and private for-profit facilities, with variable sizes, locations, and staff mixes. However, compliance with infection prevention and control practices varied very little by facility characteristics. Compliance patterns were similar across facility level, ownership type, and location, indicating that these practices appear to be ingrained as behavioural norms within the health system. There was some evidence that compliance varied by health worker characteristics. Most notably, older health workers were less likely to use gloves correctly, female health workers were better at hand hygiene, and nurses and midwives performed substantially better than more qualified providers in most of the infection prevention and control domains. From a policy perspective, these data indicate what types of health worker could be prioritised in the design and targeting of infection prevention and control interventions. Given that hospitals in our sample typically served three to four times more outpatients than health centres and dispensaries did, policy makers should also consider targeting high-volume health facilities first to maximise the number of patients benefiting initially.

No publicly owned facilities were included in the study. The most recent Service Provision Assessment in Tanzania (2014–15) found that infrastructure and supplies for infection control were often poorer in public facilities than in private facilities.^22^ For example, supplies for hand hygiene (soap and running water or alcohol-based hand disinfectant) were available in 90% of faith-based facilities, 82% of private for-profit facilities, and only 58% of government facilities. Together with the lack of variation in compliance across facilities, this leads us to expect that public sector compliance is unlikely to be higher than the rates observed in this study. This expectation is further supported by the fact that infection prevention and control compliance in our study was slightly but consistently higher than a comparable study of outpatient care in Kenya (appendix p 3), which included public, faith-based, and for-profit facilities, and found a weak negative association between public ownership and compliance.^18^

One might expect infection prevention and control compliance to be higher in inpatient settings where perceived infection risk could be higher. Studies of labour wards have shown substantial variation, with somewhat higher hand hygiene compliance in some settings (eg, 9·6% in Zanzibar)^23^ but lower in others (eg, 0·6% pre-intervention in India).^24^ In fact, non-compliance with hand hygiene has been described as a universal problem, with a 2009 systematic review in industrialised country hospitals (mainly intensive care units) reporting a median compliance rate of 40%,^25^ and much lower rates in high-income outpatient settings.^26^

This study had a number of limitations. First, because the data were originally collected for a different purpose before the pandemic, we did not measure certain supplies or behaviours crucial for the control of COVID-19, such as the wearing of personal protective equipment required to manage suspected COVID-19 patients. Second, we do not claim that our estimates are representative of private facilities in Tanzania. The sample did not include facilities in certain regions, private for-profit hospitals, or any tertiary hospitals. Moreover, we did not select a random sample among those eligible to participate. However, the lack of variation in compliance by facility level, ownership, and location gives us reason to expect a similar pattern of results elsewhere in the country. Third, by observing infection prevention and control compliance, we might have changed provider behaviour through the Hawthorne effect. However, this measurement method is likely to be more reliable than self-reports of behaviour, and our analysis of the order number of patients observed provided no strong evidence of a Hawthorne effect, although it has been noted in other studies.^20^ Fourth, we used endline data from a randomised controlled trial of a quality-improvement programme. As noted above, there was no evidence of a difference in infection prevention and control compliance between intervention and control facilities, but structural quality was assessed in both study groups at baseline by study partners, and it is possible that this could have raised awareness of infection prevention and control requirements. If awareness had been raised, it would imply that the inadequate infection prevention and control compliance observed should be considered a maximum. Fifth, we did not measure item-specific availability of infrastructure and supplies needed for infection prevention and control.

The results raise a number of crucial areas of concern. First, inadequate infection prevention and control will make health workers more likely to contract COVID-19 from their patients. Several small studies during the severe acute respiratory syndrome (SARS) epidemic, caused by another coronavirus, reported attack rates for SARS among health workers ranging from 1·2% to 57·1%.^27^, ^28^ These same health workers will be needed as front-line staff to deal with seriously ill patients as cases mount, so having significant numbers of health workers sick or self-isolating could threaten the operation of the health system at a crucial time. Second, inadequate infection prevention and control could put patients and caregivers at risk of contracting COVID-19 when they visit a health facility for another complaint. Although data on health-care-associated infections are poorly recorded in LMICs, the available evidence indicates that they are relatively common in LMIC health systems (where 15·5 patients per 100 acquire an infection, compared with 7·1 per 100 in Europe).^12^ Any such health-care-acquired infection should be avoided, but this is particularly important for COVID-19, as the case fatality rate is significantly higher for those with pre-existing conditions (eg, cardiovascular disease, diabetes, chronic respiratory disease, or cancer), who might be more likely to be visiting health facilities.

Our findings raise the question of what can be done about poor compliance. There is consensus that interventions must be based on an analysis of the causes of poor infection prevention and control in specific contexts, and that successful interventions are generally multifaceted.^29^ A systematic review of the WHO-5 hand hygiene campaign—consisting of five components, namely system change, training and education, observation and feedback, reminders, and a safety climate—found it to be effective in improving hand hygiene in hospitals, and found that compliance was further improved by adding behavioural interventions such as goal setting, reward incentives, and accountability.^30^ This review focused only on hand hygiene, and included studies were predominantly from intensive care units or other inpatient settings, with the vast majority in high-income countries. In weaker health systems, the availability of supplies is likely to be an important bottleneck. A recent WHO–UNICEF report on water, sanitation, and hygiene in health care reported that only 74% of facilities globally had basic water services (51% in sub-Saharan Africa), and 16% of facilities had no hygiene service (ie, no hand hygiene facilities at points of care and soap and water near toilets).^31^ During outbreaks, when patient numbers are likely to substantially increase, maintaining supplies will be particularly important. However, even in weaker health systems, supplies are necessary but not sufficient for infection prevention and control compliance, as shown in a large study of infection prevention and control in Kenya.^18^ In addition to the behavioural interventions highlighted above, attention should also be given to the health-system-level determinants of poor infection prevention and control, such as leadership, management, logistics, and accountability, which merit focus in future research.

The costs of compliance must also be considered. We estimate that for 1000 outpatients (the mean per month in sampled facilities was 861), the supplies needed for hand hygiene, disinfection, and waste management cost approximately US$60 in Tanzania based on current prices (assuming 4000 hand hygiene indications requiring 4 L hand soap and 4000 paper towels, 1500 gloves, 620 disinfections requiring 0·12 L disinfectant, and 50 waste bags). However, prices are rising rapidly because of increased demand during COVID-19, especially for hand rub, which is unlikely to be available in sufficient quantities on the open market. WHO recommends two formulations for local production of hand rub, but countries will need support to do this on a large scale, with proper quality assessment.^14^

To these supply costs, one would need to add costs for resources for additional training and behaviour-change activities to support implementation. Moreover, infection prevention and control for COVID-19 will require many more actions than those assessed here, particularly for inpatient care. For example, WHO COVID-19 guidelines^7^ require cleaning and disinfection of toilets at least twice daily by a trained cleaner wearing personal protective equipment (gown, gloves, boots, mask, and a face shield or goggles); once daily cleaning of all environments in which patients with COVID-19 receive care, as well as cleaning when a patient is discharged; and wearing of appropriate personal protective equipment by all individuals dealing with soiled bedding, towels, and clothes from patients with COVID-19, including heavy-duty gloves, a mask, eye protection (goggles or a face shield), a long-sleeved gown or apron, and boots or closed shoes.^7^ The extremely inadequate availability of personal protective equipment in these health systems represents a major threat.

These data highlight the massive task ahead of us in addressing nosocomial transmission of COVID-19 in a context of low baseline compliance with infection prevention and control practices. A huge injection of infection prevention and control supplies is urgently needed to cover both outpatient and inpatient care in public and private facilities. In addition, support is required for interventions that are targeted to these specific contexts and that consider behavioural determinants and the reality of facility operation under the current crisis. Fulfilling these needs will require not only national efforts, but also large-scale international collaboration and solidarity. Addressing these practices under the impetus of COVID-19 could prevent transmission of other infectious diseases within health facilities, and could even improve the culture of infection prevention and control in the long term.
