# Supplementary material for: Infection prevention and control compliance in Tanzanian outpatient facilities: a cross-sectional study with implications for the control of COVID-19
Source: Lancet Glob Health. 2020 May 6;8(6):e780–9. doi: 10.1016/S2214-109X(20)30222-9 (PMC7202838; doi:10.1016/S2214-109X(20)30222-9)
Supplement: Supplementary appendix [file mmc1.pdf]

THE LANCET
Global Health

Supplementary appendix

This appendix formed part of the original submission and has been peer reviewed. We post it as supplied by the authors.

Supplement to: Powell-Jackson T, King JJC, Makungu C, et al. Infection prevention and control compliance in Tanzanian outpatient facilities: a cross-sectional study with implications for the control of COVID-19. *Lancet Glob Health* 2020; published online May 6. http://dx.doi.org/10.1016/S2214-109X(20)30222-9.

Online appendix

Infection prevention and control compliance in Tanzanian outpatient facilities: a cross-sectional study with implications for the control of COVID-19

Timothy Powell Jackson, PhD, LSHTM, 15-17 Tavistock Place, London WC1H 9SH

Jessica J C King, MSc, LSHTM, 15-17 Tavistock Place, London WC1H 9SH

Christina Makungu, MA, Ifakara Health Institute, Mikocheni B, Kiko Avenue, P.O BOX 78373, Dar es Salaam, Tanzania

Nicole Spieker, PhD, PharmAccess. AHTC, Tower 4C, Paasheuvelweg 25, 1105 BP Amsterdam, The Netherlands

Susannah Woodd, MSc, LSHTM, Keppel Street, London WC1E 7HT, UK

Peter Risha, PhD, PharmAccess Tanzania, Ohio/Sokoine Drive 149/32 Dar es Salaam, Tanzania

Catherine Goodman, PhD, LSHTM, 15-17 Tavistock Place, London WC1H 9SH

Re-weighting sensitivity analysis

In the selection of the study health facilities, we implicitly oversampled certain types of facility (by ownership and level). As a sensitivity analysis, we re-weighted the data to account for the oversampling using information on the universe of health facilities in the country. Table A1 shows the number and percentage of facilities by ownership and level in the population and in our study sample. Using this information, we generated a probability weight, also shown in Table A1.

The sensitivity analysis involved running weighted regressions of the same models as described in the main paper. Since the analysis was conducted at the level of indication and done separately for each domain, the facility weight was divided by the number of indications observed within each domain. Table A2 reports the results of this sensitivity analysis. The findings were qualitatively similar to those reported in the main paper when we weighted the data to account for oversampling with respect to facility level and ownership.

Hawthorne effect sensitivity analysis

Directly observing health workers gives rise to the possibility that they alter their behaviour. The specific concern in our study is that health workers temporarily increased IPC compliance when they were observed by the research team, above their usual level of performance. We assessed the presence of a Hawthorne effect by examining whether compliance with IPC practices was associated with order number of patients observed. The idea behind the sensitivity analysis is that, if there was a Hawthorne effect, health workers would eventually get used to being observed and IPC compliance would revert to a lower level closer to actual practice. In the data this would show up as a negative association between IPC compliance and order of patient observation.

We analysed data at the level of patient observation. The dependent variable was IPC compliance, defined as the proportion of indications for which the health worker complied with the correct action. The independent variable of interest was the order number of patients observed. Each health worker was observed for an average of 4.9 patient interactions (SD 4.2). We ran four OLS regression models. Model 1 included no other covariates. Model 2 included an indicator for each IPC indication. Model 3 additionally controlled for facility and health worker characteristics (the same as in the main paper). Model 4 replaced the facility and health worker characteristics with health worker fixed effects, and is our preferred model.

Figure A2 shows IPC compliance by order of patient observation. IPC compliance beyond the 15th patient observation was not reported because the sample size was less than 30 thereafter. There is some variation in mean IPC compliance but essentially the line is flat, suggesting no strong association between compliance and order of patient observation. Table A3 reports the regression

results. Across the four models, there is no consistent direction in the association but the coefficient is small in magnitude in all instances. In the unadjusted model, the coefficient is positive but insignificant (p=0.241). In our preferred model with indicators for each indications and health worker fixed effects, the coefficient is again positive but insignificant (p=0.658). In models 2 and 3, the coefficient is significant, but the magnitude suggests a weak association. In model 3, for example, an increase of one in patient observation order is associated with a reduction of 0.2 percentage points in mean IPC compliance.

Other sensitivity analyses

Table A4 reports the unadjusted results, showing the correlation between IPC compliance and the characteristics of health facilities and health workers. It reports the p value from a Pearson's chi-squared, accounting for clustering at the facility level. Table A5 reports results in which we included in the regressions additional controls for patient age and gender. The findings were very similar to those reported in the main paper. Table A6 reports results for a different definition of compliance with infectious waste management – one that is less stringent than national guidelines in Tanzania – in which we no longer required the bag and bin colour to match for red and yellow bins. There were some differences in the findings with respect to health facility ownership / level and health worker age.

Comparison with Bedoya et al (2017) study in Kenya

For interested readers, we compared our results on IPC compliance with those reported in a study conducted in Kenya which used methods and tools that informed our study (Bedoya et al, 2017). We compared IPC compliance for each indication reported by the Kenya study (Table A7). To aide interpretation, we note a number of methodological differences between the two studies. First, while both studies included faith-based and private for-profit facilities, the study in Kenya also included public health facilities, but our study in Tanzania did not. Second, there were differences in the definition of a small number of indications (see the notes of the table). Third, the categorisation of indications by domain was not the same in the studies, which is why we compare IPC compliance by indication, rather than domain.

Panel A1. IPC compliance observation tool

IFAKARA HEALTH INSTITUTE research | training | services

PharmAccess FOUNDATION

LONDON SCHOOL of HYGIENE &TROPICAL MEDICINE

A STUDY TO EVALUATE THE EFFECTIVENESS OF THE "SAFECARE" APPROACH IN TANZANIA

Observation Tool

A: GENERAL DETAILS (ALL SITES)

1. Field Interviewer ID: ____ ____

2. Facility ID: ____ ____ ____

3. Date (DD/MM/YY): ____ ____/____ ____/____ ____

4. Site: □ Consultation room □ Injection room □ Dressing room □ Laboratory

5. HCW ID: ____ ____

6. Patient consent: □ Yes □ No

7. Red bin in room: □ Yes □ No

8. Red bag in red bin: □ Yes □ No

9. Yellow bin in room: □ Yes □ No

10. Yellow bag in yellow bin: □ Yes □ No

11. Start time: :

12. End time :

13. Patient ID: ____ ____ ____ ____

14. Patient gender: □ Male □ Female

15. Patient age (approximate): □ <5 □ 5-17 □ 18-29 □ 30-49 □ 50+

16. Observation result: □ Completed □ Partially completed

17. If partially completed, give reason: □ Ended by provider □ Ended by patient □ Ended by interviewer □ Other (specify): ____________________

B: HAND HYGIENE (CONSULTATION OR DRESSING)

18. Are gloves used?
Yes □
No □

If yes:
19. Are gloves.... □ New □ Reused □ Cannot say
20. Hand hygiene directly before gloves: □ HR □ HW w/soap □ HW no soap □ None □ Cannot say
21. Hand hygiene directly after gloves: □ HR □ HW w/soap □ HW no soap □ None □ Cannot say

22. Patient contact or exam (tick all that apply):
□ Touching skin □ Cleaning body fluids
□ Mouth or throat exam □ Nose exam
□ Ear exam □ Eye exam
□ Wound cleaning or dressing □ Wound exam
□ Stiches □ Preparation of medicine
□ Inserting a suppository □ Vaginal, male genital or rectal exam
□ Listening to chest □ Handling container of body fluid
□ Taking temperature

□ Other (specify): ____________________

□ No patient contact and no exam (skip to 31)

23. Hand hygiene before contact or exam:
□ HR
□ HW w/soap
□ HW no soap
□ None
□ Cannot say

If yes: 24. Hand hygiene took _______ seconds

25. Hand hygiene with gloves on:
□ Yes
□ No

26. Drying method:
□ Clean disposable towel
□ Nothing
□ Reused towel or clothes
□ Cannot say

27. Hand hygiene after contact or exam:
□ HR
□ HW w/soap
□ HW no soap
□ None
□ Cannot say

If yes: 28. Hand hygiene took _______ seconds

29. Hand hygiene with gloves on:
□ Yes
□ No

30. Drying method:
□ Clean disposable towel
□ Nothing
□ Reused towel or clothes
□ Cannot say

C: WASTE SEGREGATION AND DISINFECTION (CONSULTATION OR DRESSING)

31: Thermometer used: □ Yes □ No

If yes:
32. Type □ Standard □ Infra-red
33. Disinfection (before or after use):
□ Disinfected with rubbing alcohol/bleach
□ Not disinfected, but cleaned
□ Not disinfected, not cleaned
□ Left in disinfectant
□ Cannot say

34. Stethoscope used: □ Yes □ No

If yes:
35. Disinfection (before or after use):
□ Disinfected with rubbing alcohol/bleach
□ Not disinfected, but cleaned
□ Not disinfected, not cleaned
□ Cannot say

36. Tongue depressor used: □ Yes □ No

If yes:
37. Type: □ Plastic □ Wooden □ Metallic
38. Segregated....
□ Black or blue bin □ Yellow bin
□ Red bin □ Other bin
□ Disinfected with rubbing alcohol/bleach
□ Not disinfected, but cleaned
□ Not disinfected, not cleaned
□ Cannot say

39. Otoscope used: □ Yes □ No

If yes:
40. Disinfection (before or after use):
□ Disinfected with rubbing alcohol/bleach
□ Not disinfected, but cleaned
□ Not disinfected, not cleaned
□ Cannot say

41. Gloves used: □ Yes □ No

42. If yes:
Segregated....
□ Black or blue bin □ Yellow bin
□ Red bin □ Other bin
□ Left on hands
□ Left outside
□ Cannot say

43. Swabs/gauze/bandages used on patient: □ Yes □ No

44. If yes:
Segregated....
□ Black or blue bin □ Yellow bin
□ Red bin □ Other bin
□ Left outside
□ Cannot say

D: INJECTION/BLOOD DRAW

45. Injection: □ Intravenous (into vein) □ Intramuscular, intradermal or subcutaneous

46. Blood draw: □ From vein □ Finger or heel prick

E: DISINFECTION/PREPARATION/SEGREGATION

47. Lancet used:
□ Yes
□ No

If used:
48. Source:
□ New (original package)
□ Reused
□ Cannot say

49. Disposal
Segregated …
□ Safety sharps container
□ Improvised sharps container
□ Segregated in other bin
□ Stored for sterilisation
□ Left outside
□ Cannot say

50. Needle used:
□ Syringe
□ Vacutainer
□ Butterfly
□ Other
□ None

If used:
51. Source:
□ New (original package)
□ Reused
□ Cannot say

52. Disposal:
Segregated …
□ Safety sharps container
□ Improvised sharps container
□ Segregated in other bin
□ Stored for sterilisation
□ Left outside
□ Cannot say

53. Capillary tube used:
□ Yes
□ No

If yes:
54. Segregated…
□ Safety sharps container
□ Improvised sharps container
□ Black or blue bin
□ Yellow bin
□ Red bin
□ Other bin
□ Left outside
□ Cannot say

55. Glucometer/HB strip used:
□ Yes
□ No

If yes:
56. Segregated…
□ Safety sharps container
□ Improvised sharps container
□ Black or blue bin
□ Yellow bin
□ Red bin
□ Other bin
□ Left outside
□ Cannot say

57. Work surface:
□ Clean
□ Dirty, blood
□ Dirty, used syringes/needles
□ Dirty, used swabs/gauze
□ Dirty, other infectious waste

58. Patient skin prep:
Clean swab…
□ Spirit dispenser + cotton wool
□ Spirit bottle + cotton wool
□ Wet-prep cotton wool
□ Standardised-alcohol swab
□ Dirty swab
□ None

59. Cotton wool (bloodied):
□ Yes
□ No

If yes:
60. Segregated…
□ Safety sharps container
□ Improvised sharps container
□ Black or blue bin
□ Yellow bin
□ Red bin
□ Other bin
□ Left outside or with patient
□ Cannot say

61. Gloves used:
□ Yes
□ No

If yes:
62. Segregated…
□ Safety sharps container
□ Improvised sharps container
□ Black or blue bin
□ Yellow bin
□ Red bin
□ Other bin
□ Left on hands
□ Left outside
□ Cannot say

63. Needle/lancet recapped after use:
□ No
□ One hand
□ Two hands

64. Needle separated:
□ No
□ Removal device
□ Hands

65. HCW had needle/lancet-prick:
□ No
□ Yes

F: HAND HYGIENE

66. Are gloves used? Yes □ No □

If yes:

67. Are gloves…
□ New □ Reused □ Cannot say

68. HH directly before gloves:
□ HR
□ HW w/soap
□ HW no soap
□ None
□ Cannot say

69. HH directly after gloves:
□ HR
□ HW w/soap
□ HW no soap
□ None
□ Cannot say

70. Hand hygiene before procedure:
□ HR
□ HW w/soap
□ HW no soap
□ None
□ Cannot say

71: Hand hygiene took
If yes: _______ seconds

72: Hand hygiene with gloves on:
□ Yes
□ No

73: Drying method:
□ Clean disposable towel
□ Nothing
□ Reused towel or clothes
□ Cannot say

74. Hand hygiene after procedure:
□ HR
□ HW w/soap
□ HW no soap
□ None
□ Cannot say

75. Hand hygiene took
If yes: _______ seconds

76. Hand hygiene with gloves on:
□ Yes
□ No

77. Drying method:
□ Clean disposable towel
□ Nothing
□ Reused towel or clothes
□ Cannot say

Figure A1. Compliance with IPC practices for injection and blood draw safety

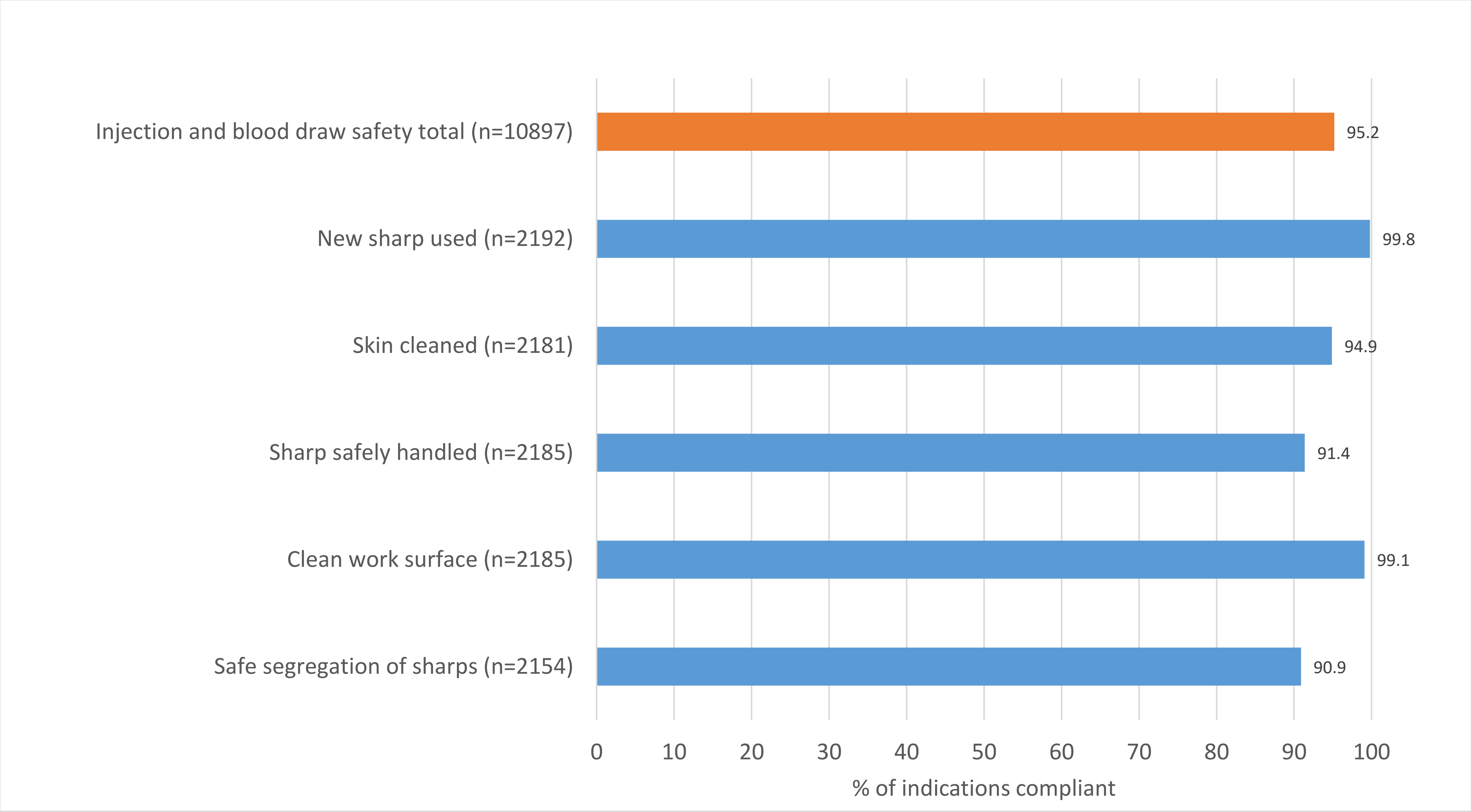

Figure A2. IPC compliance and order of patient observation

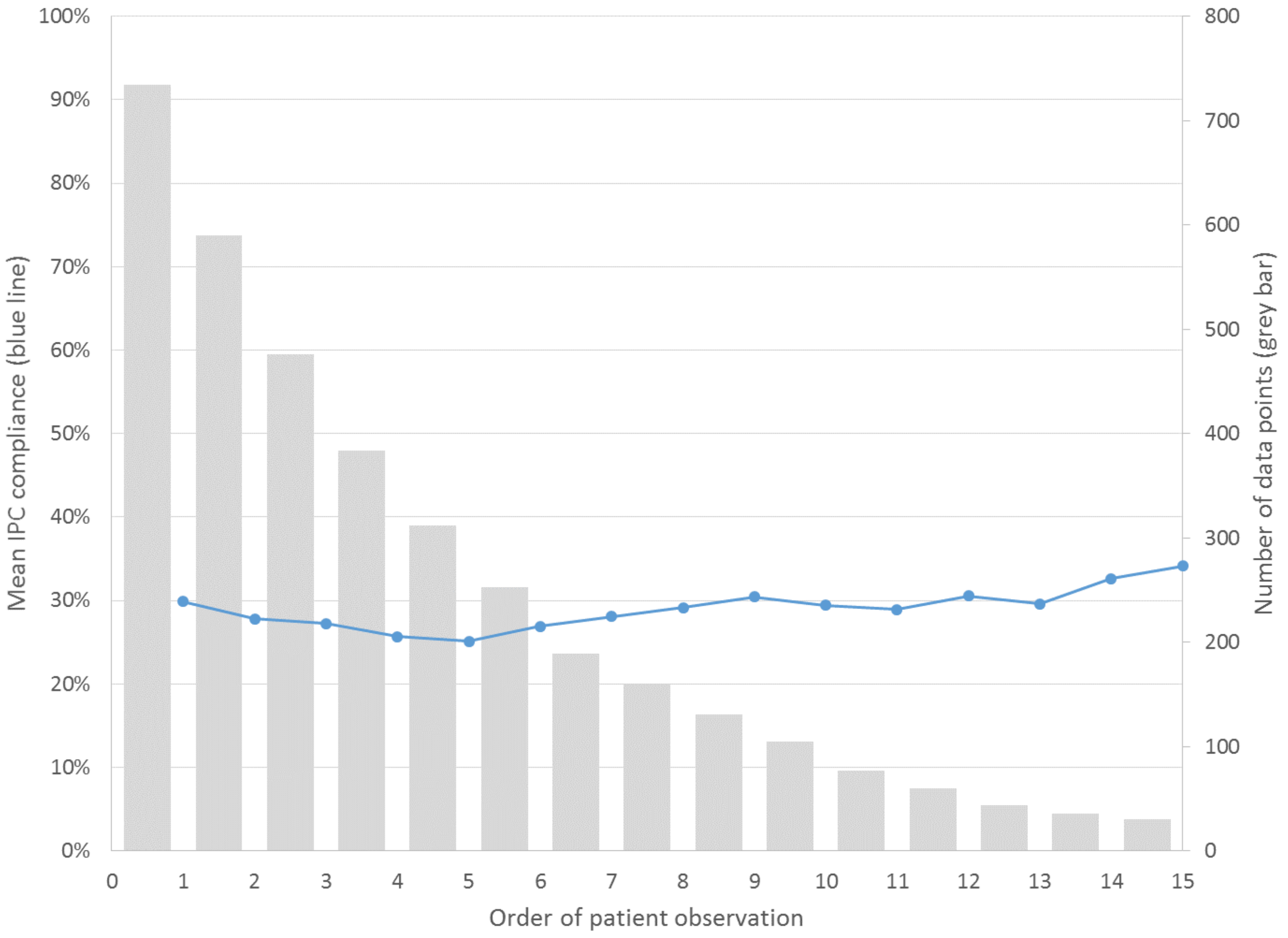

Table A1. Calculation of survey weights

	Population of facilities (A)		Study facilities (B)		Facility weight (A/B)
	N	%	N	%	
Private for-profit dispensaries	755	43%	79	36%	9.56
Private for-profit health centres	109	6%	19	9%	5.74
FBO dispensaries	658	37%	39	18%	16.87
FBO health centres	150	8%	47	21%	3.19
FBO hospitals	100	6%	36	16%	2.78
Total	1772		220		8.05

Table A2. Multilevel mixed effects logistic regressions with survey weights

	Hand hygiene [N=8,651]			Glove use [N=4,912]			Disinfection of reusable equipment [N=751]			Waste management [N=4,296]		
	%	OR	P value	%	OR	P value	%	OR	P value	%	OR	P value
**Facility characteristics**												
Facility level and ownership												
Private for-profit dispensaries	7.5	Reference		72.2	Reference		3.3	Reference		47.9	Reference	
Private for-profit health centres	8.4	1.22 (0.52 to 2.87)	0.654	66.7	0.89 (0.35 to 2.86)	0.810	0	NS		41.9	0.79 (0.30 to 2.08)	0.640
FBO dispensaries	7.9	0.62 (0.25 to 1.51)	0.294	72.6	1.75 (0.88 to 3.49)	0.113	2.6	2.84 (0.18 to 44.3)	0.456	43.5	0.53 (0.25 to 1.13)	0.100
FBO health centres	9.7	1.21 (0.55 to 2.67)	0.635	65.6	0.70 (0.36 to 1.37)	0.300	4.4	4.76 (0.17 to 135)	0.360	35.8	0.30 (0.14 to 0.67)	0.003
FBO hospitals	3.9	0.54 (0.24 to 1.21)	0.132	76.0	1.11 (0.62 to 1.97)	0.721	4.9	382.57 (0.15 to >1000)	0.136	40.3	0.35 (0.14 to 0.85)	0.021
Facility location												
Dar es Salaam	5.9	Reference		76.8	Reference		3.6	Reference		48.4	Reference	
Other urban/peri-urban	7.3	1.40 (0.67 to 2.93)	0.365	69.8	0.79 (0.42 to 1.50)	0.476	4.4	4.24 (0.49 to 39.96)	0.191	41.2	0.63 (0.32 to 1.25)	0.186
Rural	9.2	1.58 (0.60 to 4.15)	0.358	70.8	0.78 (0.38 to 1.60)	0.498	1.2	0.09 (0.0004 to 15.43)	0.355	45.9	1.22 (0.54 to 2.78)	0.634
**Staff characteristics**												
Age (years)												
<30	7.1	Reference		80.3	Reference		3.2	Reference		45.0	Reference	
30-49	8.5	0.96 (0.55 to 1.65)	0.869	73.4	0.50 (0.29 to 0.88)	0.017	5.3	2.41 (0.21 to 27.98)	0.482	44.8	1.18 (0.76 to 1.83)	0.462
≥50	7.8	1.00 (0.56 to 1.78)	0.988	41.0	0.25 (0.13 to 0.49)	<0.001	1.1	0.01 (0 to 344)	0.408	41.3	0.93 (0.47 to 1.86)	0.350
Gender												
Male	4.9	Reference		68.1	Reference		2.8	Reference		45.5	Reference	
Female	11.6	2.12 (1.25 to 3.60)	0.005	75.2	0.86 (0.50 to 1.45)	0.567	4.4	6.63 (0.06 to 689)	0.424	43.6	1.22 (0.80 to 1.86)	0.350
Cadre												
Medical doctor	4.4	0.94 (0.38 to 2.35)	0.902	29.2	0.96 (0.27 to 3.34)	0.946	4.1	0.37 (0.01 to 37.76)	0.558	53.4	0.40 (0.10 to 1.69)	0.213
Assistant medical officer/clinical officer	6.3	Reference		29.9	Reference		2.7	Reference		58.3	Reference	
Nurse/midwife	28.4	3.92 (2.03 to 7.56)	<0.001	76.2	5.94 (2.35 to 14.99)	<0.001	0	NS		52.0	0.94 (0.27 to 3.23)	0.918
Nursing/medical assistant	7.6	0.71 (0.33 to 1.53)	0.386	65.8	4.36 (1.55 to 12.26)	0.005	13.5	2.07 (0.11 to 38.76)	0.626	39.1	0.43 (0.10 to 1.74)	0.234
Laboratory technician/assistant	3.8	0.27 (0.13 to 0.55)	<0.001	84.3	14.22 (7.20 to 28.01)	<0.001	3.7	8.38 (0.17 to 405)	0.283	43.4	0.27 (0.o8 to 0.94)	0.040

Table A3. Testing for a Hawthorne effect: is compliance associated with order number of patients observed?

	Model 1: unadjusted		Model 2: IPC indication		Model 3: facility and health worker characteristics		Model 4: health worker fixed effects	
	Coefficient (95%CI)	**P value**	**Coefficient (95%CI)**	**P value**	**Coefficient (95%CI)**	**P value**	**Coefficient (95%CI)**	**P value**
Order number of patients observed	0.002 (-0.002 to 0.006)	0.241	-0.005 (-0.007 to -0.002)	<0.001	-0.002 (-0.005 to 0)	0.050	0.0004 (-0.001 to 0.002)	0.658
Number of observations	3686		3686		3686		3686	
Number of facilities	220		220		220		220	

Notes: Table shows results from OLS regressions in which standard errors are clustered at the level of facility. Data are analysed at the level of patient observation. The dependent variable is IPC compliance defined as the proportion of indications for which the health worker complied with the correct action. The independent variable of interest is the order number of patients observed. Each health worker was observed for an average of 4.9 patient interactions (SD 4.2). Model 1 includes no other covariates. Model 2 includes an indicator for each IPC indication. Model 3 additionally controls for facility and health worker characteristics. Model 4 replaces the facility and health worker characteristics with health worker fixed effects.

Table A4. Unadjusted results

	Hand hygiene [N=8,651]		Glove use [N=4,912]		Disinfection of reusable equipment [N=751]		Waste management [N=4,296]	
	%	P value	%	P value	%	P value	%	P value
**Facility characteristics**								
Facility level and ownership								
Private for-profit dispensaries	5.3		75.5		4.6		49.1	
Private for-profit health centres	8.5	0.0180	71.4	0.3878	0.0	0.5817	41.4	0.0678
FBO dispensaries	6.3		79.8		3.2		49.0	
FBO health centres	10.2		71.0		5.6		36.6	
FBO hospitals	4.2		75.8		8.1		40.1	
Facility location								
Dar es Salaam	6.5		79.4		5.0		49.2	
Other urban/peri-urban	6.9	0.9743	73.0	0.2369	6.0	0.9529	40.2	0.1540
Rural	6.9		75.0		4.8		44.4	
**Staff characteristics**								
Age (years)								
<30	6.8		81.1		7.2		45.4	
30-49	6.9	0.9947	76.0	<0.0001	7.9	0.1695	43.7	0.1249
≥50	6.9		52.6		2.2		33.7	
Gender								
Male	4.9	0.0001	71.8	0.0100	4.8	0.3962	42.2	0.4421
Female	10.2		78.7		8.1		44.6	
Cadre								
Medical doctor	8.7		29.1		6.9		NS	
Assistant medical officer/clinical officer	6.2		32.6		4.9		62.6	
Nurse/midwife	22.2	<0.0001	81.7	<0.0001	NS	0.8093	54.2	0.0004
Nursing/medical assistant	13.0		76.0		NS		45.1	
Laboratory technician/assistant	2.3		82.7		NS		40.3	

Table A5. Multilevel mixed effects logistic regressions, adjusting for patient characteristics (not reported)

	Hand hygiene [N=8,651]			Glove use [N=4,912]			Disinfection of reusable equipment [N=751]			Waste management [N=4,296]		
	%	OR	P value	%	OR	P value	%	OR	P value	%	OR	P value
**Facility characteristics**												
Facility level and ownership												
Private for-profit dispensaries	5.3	Reference		75.5	Reference		4.6	Reference		49.1	Reference	
Private for-profit health centres	8.5	1.65 (0.58 – 4.70)	0.352	71.4	1.03 (0.48– 2.21)	0.942	0.0	-	-	41.4	0.74 (0.31 – 1.76)	0.493
FBO dispensaries	6.3	0.84 (0.33 – 2.13)	0.719	79.8	1.63 (0.85 – 3.13)	0.139	3.2	1.99 (0.07 –54.63)	0.683	49.0	0.74 (0.35 – 1.55)	0.420
FBO health centres	10.2	1.87 (0.80 – 4.34)	0.146	71.0	0.82 (0.45 – 1.49)	0.511	5.6	1.72 (0.07 –44.14)	0.743	36.6	0.36 (0.18 – 0.71)	0.004
FBO hospitals	4.2	0.86 (0.34 – 2.16)	0.749	75.8	1.32 (0.70 – 2.48)	0.395	8.1	15.32 (0.43 – 548.16)	0.135	40.1	0.47 (0.23 – 0.96)	0.038
Facility location												
Dar es Salaam	6.5	Reference		79.4	Reference		5.0	Reference		49.2	Reference	
Other urban/peri-urban	6.9	1.22 (0.54 – 2.76)	0.635	73.0	0.74 (0.41 – 1.31)	0.299	6.0	3.17 (0.15 – 67.36)	0.460	40.2	0.67 (0.35 – 1.28)	0.219
Rural	6.9	0.87 (0.34 – 2.22)	0.768	75.0	0.64 (0.33 – 1.25)	0.193	4.8	0.36 (0.01- 14.7)	0.589	44.4	1.14 (0.54 – 2.40)	0.727
**Staff characteristics**												
Age (years)												
<30	6.8	Reference		81.1	Reference		7.2	Reference		45.4	Reference	
30-49	6.9	1.08 (0.80 – 1.45)	0.621	76.0	0.64 (0.50 – 0.82)	<0.001	7.9	2.55 (0.24 – 27.36)	0.440	43.7	0. 83 (0.62 – 1.09)	0.182
≥50	6.9	0.91 (0.64 – 1.29)	0.605	52.6	0.33 (0.24 – 0.46)	<0.001	2.2	0.08 (0.00 – 1.61)	0.100	33.7	0. 74 (0.48 – 1.15)	0.185
Gender												
Male	4.9	Reference		71.8	Reference		4.8	Reference		42.2	Reference	
Female	10.2	1.98 (1.50 – 2.60)	<0.001	78.7	0.89 (0.70 – 1.14)	0.370	8.1	2.42 (0.19 – 30.30)	0.492	44.6	1.01 (0.77-1.33)	0.945
Cadre												
Medical doctor	8.7	1.20 (0.73 – 1.98)	0.464	29.1	0.59 (0.33 – 1.06)	0.077	6.9	1.15 (0.05 – 25.85)	0.928	NS	NS	NS
Assistant medical officer/clinical officer	6.2	Reference		32.6	Reference		4.9	Reference		62.6	Reference	
Nurse/midwife	22.2	6.01 (4.03 – 8.96)	<0.001	81.7	10.32 (6.84 – 15.55)	<0.001	NS	NS	NS	54.2	0.82 (0.44 – 1.52)	0.524
Nursing/medical assistant	13.0	2.65 (1.66 – 4.22)	<0.001	76.0	6.03 (4.11 – 8.86)	<0.001	NS	NS	NS	45.1	0.58 (0.32 – 1.06)	0.076
Laboratory technician/assistant	2.3	0.27 (0.16 – 0.43)	<0.001	82.7	12.13 (9.11– 16.15)	<0.001	NS	NS	NS	40.3	0.25 (0.14 – 0.44)	<0.001

Table A6. Multilevel mixed effects logistic regressions with varying definitions of compliance for infectious waste management

	Requiring bag and bin colour to match for red and yellow bins (as presented in main paper) [N=4,296]			Not requiring bag and bin colour to match for red and yellow bins [N=4,296]		
	**%**	**OR**	**P value**	**%**	**OR**	**P value**
**Facility characteristics**						
Facility level and ownership						
Private for-profit dispensaries	49.1	Reference		62.2	Reference	
Private for-profit health centres	41.4	0.74 (0.31 – 1.77)	0.498	60.7	0.71 (0.32 – 1.57)	0.397
FBO dispensaries	49.0	0.74 (0.35 – 1.55)	0.420	64.9	1.08 (0.53 – 2.15)	0.826
FBO health centres	36.6	0.36 (0.18 – 0.72)	0.004	55.6	0.71 (0.37 – 1.34)	0.291
FBO hospitals	40.1	0.46 (0.22 – 0.95)	0.037	55.9	0.61 (0.31 – 1.20)	0.151
Facility location						
Dar es Salaam	49.2	Reference		59.9	Reference	
Other urban/peri-urban	40.2	0.66 (0.34 – 1.26)	0.210	58.7	1.29 (0.71 – 2.34)	0.409
Rural	44.4	1.13 (0.54 – 2.38)	0.741	59.0	1.52 (0.76 – 3.02)	0.234
**Staff characteristics**						
Age (years)						
<30	45.4	Reference		61.7	Reference	
30-49	43.7	0. 83 (0.62 – 1.09)	0.182	59.9	0. 82 (0.42 – 1.04)	0.107
≥50	33.7	0. 74 (0.48 – 1.15)	0.185	47.9	0. 62 (0.42 – 0.92)	0.016
Gender						
Male	42.2	Reference		57.9	Reference	
Female	44.6	1.01 (0.77-1.33)	0.945	61.0	0.92 (0.73-1.17)	0.495
Cadre						
Medical doctor	NS	NS	NS	NS	NS	NS
Assistant medical officer/clinical officer	62.6	Reference		73.0	Reference	
Nurse/midwife	54.2	0.82 (0.44 – 1.52)	0.524	71.3	0.70 (0.41 – 1.20)	0.198
Nursing/medical assistant	45.1	0.58 (0.32 – 1.06)	0.076	60.0	0.63 (0.37 – 1.07)	0.085
Laboratory technician/assistant	40.3	0.25 (0.14 – 0.44)	<0.001	56.5	0.36 (0.21 – 0.55)	<0.001

Table A7. Comparison of IPC compliance in Tanzania and Kenya

Indication	Kenya (Bedoya et al, 2017)		Tanzania (this study)	
	N	Compliance	N	Compliance
Hand hygiene before touching a patient	8062	1.5%	1464	4.4%
Hand hygiene after touching a patient	8073	1.6%	1464	9.2%
Hand hygiene before a clean/aseptic procedure	1187	2.7%	680	7.1%
Hand hygiene after exposure to body fluids	1021	6.8%	677	13.4%
Hand hygiene after contact with patient surroundings	13599	0.7%		
Hand hygiene before injection/blood draw procedure	7393	1.9%	2185	3.4%
Hand hygiene after injection/blood draw procedure	7369	6.4%	2185	8.3%
New gloves are used for each patient	3633	63.9%	2043	80.0%
Hand hygiene is performed after wearing gloves	3658	5.4%	1788	3.6%
Health workers wear gloves for potential contact with blood, body fluids, mucous membranes	9383	39.8%	2872[1]	71.1%
Gloves are removed and discarded after patient interaction/before leaving care area	3635	56.9%	1993	72.2%
Needles are used for only one patient	7235	99.9%	2192[2]	99.8%
Syringes are used for only one patient	2952	99.1%		
Skin is prepared using aseptic procedure	7437	69.8%	2181	94.9%
Health workers disinfect (standard) thermometers after patient use	1302	29.5%	224[3]	13.0%
Health workers disinfect stethoscopes after patient use	1622	2.8%	579[3]	0.7%
Syringes segregated in a puncture-resistant sharps container	2856	73.5%		
Needle segregated in puncture-resistant sharps container	7201	85.2%	2154[2]	90.9%
Health workers segregate other medical waste related to injections and blood draws, including swabs/gauze and excluding syringes and needles and gloves, into the corresponding red or yellow color-coded bins with matching bags	7010	6.8%	2120[4]	18.7%
Health workers segregate other medical waste related to patient examination, including swabs/gauze into the corresponding red or yellow color-coded bins with matching bags if it is infectious in nature	1836	0.3%	186	13.4%

Notes: 1. This combines two indications presented in the main paper. 2. This number includes all sharps (needles and lancets) 3. In our Tanzania study, this indication was also coded as compliant if disinfected before patient use 4. In our Tanzania study, this indication was also coded as compliant if segregated into the sharps container.
